# Enzymatic hydrolysis of biomass from wood

**DOI:** 10.1111/1751-7915.12346

**Published:** 2016-02-01

**Authors:** Consolación Álvarez, Francisco Manuel Reyes‐Sosa, Bruno Díez

**Affiliations:** ^1^ Department of BiotechnologyAbengoa ResearchCampus Palmas Altas, C/Energía Solar no. 1Seville41014Spain

## Abstract

Current research and development in cellulosic ethanol production has been focused mainly on agricultural residues and dedicated energy crops such as corn stover and switchgrass; however, woody biomass remains a very important feedstock for ethanol production. The precise composition of hemicellulose in the wood is strongly dependent on the plant species, therefore different types of enzymes are needed based on hemicellulose complexity and type of pretreatment. In general, hardwood species have much lower recalcitrance to enzymes than softwood. For hardwood, xylanases, beta‐xylosidases and xyloglucanases are the main hemicellulases involved in degradation of the hemicellulose backbone, while for softwood the effect of mannanases and beta‐mannosidases is more relevant. Furthermore, there are different key accessory enzymes involved in removing the hemicellulosic fraction and increasing accessibility of cellulases to the cellulose fibres improving the hydrolysis process. A diversity of enzymatic cocktails has been tested using from low to high densities of biomass (2–20% total solids) and a broad range of results has been obtained. The performance of recently developed commercial cocktails on hardwoods and softwoods will enable a further step for the commercialization of fuel ethanol from wood.

## Introduction

The applied research and development for second generation of ethanol production has been focused on cellulosic materials such as energy crops or agricultural residues like switchgrass, corn stover or sugarcane straw and bagasse. However, minor attention has been dedicated to woody biomass that could be also a feasible option for cellulosic with several advantages in terms of production, harvesting, storage and transportation, compared with herbaceous biomass for bioconversion (Zhu and Pan, 2010).

Woody biomass could be sustainably obtained in large quantities from forestlands or even intensively managed worldwide plantations (Perlack *et al*., 2005). In the case of cultivated areas it usually comes from short‐rotation woody crops such as Populus, Salix, Eucalyptus, Alnus, Loblolly, lodgepole, Ponderosa among other hardwoods and softwoods strains or varieties (Johnson *et al*., 2007). Furthermore, short‐rotation woody crops and other intensively managed tree cultures are one of the most sustainable sources of biomass, provided they are strategically placed on the landscape and managed with good practices for soil and water conservation, nutrients recycling and suitable genetic diversity maintenance (Hall, 2008). These woody biomass sources also provide secondary benefits such as carbon sequestration, standing out as an ecosystem for wildlife and soil quality stabilization (Moser *et al*., 2002).

Hundreds of millions of tons of woody biomass, accounting for 30% of the total biomass projected to be available for biofuel, can be sustainably available in various regions of the world such as United States, Scandinavia, New Zealand, Canada, Japan and South America (Zhu and Pan, 2010; da Silva *et al*., 2013).

The major differences between woody and non‐woody (agricultural) biomass are their physical properties and chemical compositions. Woody biomass is physically designed to be resistant biomass containing more lignin than agricultural biomass, which makes it very recalcitrant to microbial destruction. Within major types of woody biomass (hardwood and softwood) some compositional differences also exist. In general, hardwood species are not as recalcitrant to the action of enzymes as the softwoods. Among others, hardwoods have higher xylan and lower mannan content than softwoods (Table 1). This analysis suggests that research efforts on woody biomass should be focused on upstream processing (e.g. wood size reduction and pretreatment) to reduce its intrinsic recalcitrance and enhance the microbial hydrolysis of its polysaccharides (Zhu and Pan, 2010).

**Table 1:** Composition of representative lignocellulosic biomass sources

	Composition (%)					
	Lignin	Arabinan	Galactan	Glucan	Xylan	Mannan
Softwood						
Spruce^a^	28.3	1.4	2.7	43.2	5.7	11.5
Lodgepole pine^a^	27.9	1.6	2.1	42.5	5.5	11.6
Ponderosa pine^a^	26.9	1.8	3.9	41.7	6.3	10.8
Douglas‐fir^b^	32	2.7	4.7	44	2.8	11
Loblolly pine^b^	28	1.7	2.3	45	6.8	11
Hardwood						
Aspen^a^	23	0	0	45.9	16.7	1.2
Salix^c^	26.4	1.2	2.3	41.4	15	3.2
Yellow poplar^d^	23.3	0.5	1	42.1	15.1	2.4
Hybrid poplar^d^	23.9	0.6	0.6	43.7	17.4	2.9
Eucalyptus saligna^d^	26.9	0.3	0.7	48.1	10.4	1.3
Non‐woody						
Corn stover^d^	20.2	2	0.7	38.1	20.3	0.4
Switch grass^d^	23.1	1.5	0.5	35.9	19.6	0.4
Wheat straw^d^	16.9	2.4	0.8	32.6	19.2	0.3

a All carbohydrate data for spruce from Zhu *et al*. (2009a); lodgepole and ponderosa pine from Youngblood *et al*. (2009); aspen from Wang *et al*.(2009).

b All carbohydrate data from Pettersen (1984).

c All data from Sassner *et al*. (2008).

d All carbohydrate data from US DOE Biomass program database, http://www1.eere.energy.gov/biomass/feedstock_database.html.

Even being such woody biomass available and sustainable, its continued usefulness should be improved selecting species adapted for cellulosic ethanol production which show energy efficiency of conversion; by the effectiveness of pretreatment technologies, reduction in specific levels of recalcitrance; and useful strategies for enhancing enzymatic saccharification (Lynd *et al*., 2008).

## Structural basis for the recalcitrance of biomass from wood

The structure of lignocellulosic material has been selected in the nature to resist the microbial degradation in order to maintain the erected structure enabling the plants to capture the sunlight. The recalcitrance of these structural materials is based on a complex polymeric structure with variable proportions of cellulose, hemicellulose and lignin. With a linearly linked structure of beta‐1,4‐D‐glucose units, cellulose is the simplest of these polymers. Nevertheless, the linear structures of cellulose bind one to another by hydrogen bonding in a tight and hierarchical manner, excluding the water and difficulting the penetration and activity of degradative enzymes. This way, the elementary fibrils of 3–5 nm of diameter bind to form microfibrils of up to 20 nm (Wegner and Jones, 2009). The microfibrils form a matrix structure bundle that forms the basic cellulose fibre in the cell walls. Hemicellulose polymers have higher structural diversity and are usually classified according to the predominant sugar in the main beta‐1,4‐linked polymeric chain. This way, the main chains of xylans are formed by D‐xylose units, while mannans are composed of D‐mannose and xyloglucans contain D‐glucose. Hemicelluloses are also branched with monomers of D‐xylose, D‐galactose, L‐arabinose and D‐glucuronic acid raising a high diversity of structures. Depending on the type of wood, different structures of the hemicellulose can be found (Table 2). The main hemicelluloses from hardwoods are glucuronoxylans (O‐acetyl‐4‐O‐methylglucuronoxylan) which may contain also small amounts of glucomannans. The structure consists in a linear backbone of beta‐(1,4) D‐xylopyranose with some of the xyloses acetylated and about one‐tenth of them carrying a uronic acid (4‐O‐methylglucuronic acid) with alpha‐(1,2) linkages (Pereira *et al*., 2003). Galactoglucomannans (O‐acetyl‐galactoglucomannans) are the predominant hemicelluloses of softwoods. They consist of a linear backbone of beta‐D‐glucopyranosyl and beta‐D‐mannopyranosyl units, linked by beta‐(1,4) glycosidic bonds, partially acetylated at C2 or C3 and substituted by alpha‐D‐galactopyranosyl units. Xyloglucans consist of a beta‐(1,4)‐D‐glucose chain with 75% of the residues carrying an O‐6 D‐xylose branch. Many xylose residues carry additional L‐arabinose and D‐galactose residues forming di‐glycosyl or tri‐glycosyl branches (de Vries and Visser, 2001).

**Table 2:** Main types of polysaccharides present in hemicelluloses (information based on Alen, 2000; de Vries and Visser, 2001; Ebringerova *et al*., 2005; Pereira *et al*., 2003)

Biological origin	Hemicellulose polymers	Linkages
Corn stover	Arabinoxylans	β‐(1,4)‐Xyl (backbone)
		ɑ‐(1,2)‐Ara, ɑ(1,3)Ara
	Arabino‐glucuronoxylans	β‐(1,4)‐Xyl (backbone)
		ɑ‐(1,2)‐Ara, ɑ(1,3)Ara
		ɑ‐(1,2)‐4‐O‐metil‐ɑ‐glucuronic
Hardwood	Glucuronoxylans	β‐(1,4)‐Xyl (backbone)
		ɑ‐(1,2)‐Acetyl, ɑ‐(1,3)‐Acetyl
		ɑ‐(1,2)‐4‐O‐metil‐ɑ‐glucuronic
	Xyloglucans	β‐(1,4)‐Glc (backbone)
		ɑ‐(1,6)‐Xyl
		ɑ‐(1,2)‐Acetyl
		ɑ‐(1,2)‐Fuc
		ɑ‐(1,2)‐Ara, ɑ‐(1,3)‐Ara
		ɑ‐(1,3)‐Gal
Softwood	Galacto‐glucomannan	β‐(1,4)‐Glc‐Man (backbone)
		ɑ‐(1,6)‐Gal
		ɑ‐(1,2)‐Acetyl, ɑ‐(1,3)‐Acetyl
	Glucomannans	β‐(1,4)‐Glc‐Man (backbone)

On the other side, lignin is an unusually complex biopolymer lacking defined primary structure and formed by aromatic alcohols known as monolignols. Lignin is considered a main defence barrier against enzymatic degradation of the wood biomass. Unproductive binding of the enzymes to the lignin has been proposed to act as a physical barrier slowing down the enzymatic degradation (Mansfield *et al*., 1999). At molecular level, the chemical structure of the functional groups of lignin can also play a relevant role in the interactions with the enzymes (Pan *et al*., 2008; Liu *et al*., 2010). Lignin chemical modification or by the selective removal of lignin active fractions have resulted in improved enzymatic hydrolysis yield (Pan *et al*., 2005, 2008; Shuai *et al*., 2010). The use of surfactants or non‐enzymatic protein (BSA) to reduce or block the physical adsorption of the hydrolytic enzymes to lignin has been reported to improve significantly the efficiency of the saccharification of lignocellulosic materials. The use of divalent metal salts like CaSO_4_, MgSO_4_ and CaCl_2_ have been also described to reduce unproductive adsorption of enzymes through the formation of lignin–metal complexes (Liu *et al*., 2010) increasing significantly the enzymatic hydrolysis of lignocellulosic materials.

**Figure 1:**
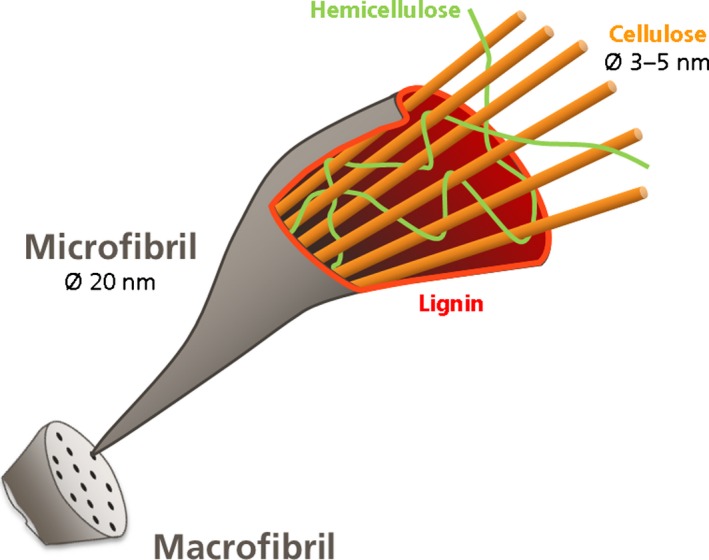
Schematic structure of a lignocellulosic biomass fibre containing cellulose, hemicellulose and lignin.

Hemicelluloses and lignin are intermingled along the hierarchical structure of cellulose, from elementary fibrils to upper fibres, filling, gluing and reinforcing the whole structure (Fig. 1). The variable chemical composition and microstructure of the biomass enable to have flexible structures like herbaceous materials or stronger and sturdier structures for wood biomass for trunks and branches of trees. The strength of these woody materials is based both on specific chemical compositions like higher lignin content and also on specific physical structures causing a higher level of recalcitrance to degradation.

Due to the lack of accessibility of this complex chemical structure of the biomass to the hydrolytic enzymes, the process of the production of ethanol from lignocellulosic biomass includes a physicochemical pretreatment. The pretreatment includes mechanical means like milling and grinding for particle size reduction followed by heat and pressure treatments (Zhu *et al*., 2009b). The pretreatment processes often include the use of chemicals to modify or remove chemical components, solubilizing lignin to various extents, hydrolysing the hemicelluloses and breaking cellulosic chemical bonds, loosening the structure of the cellulosic fibres for a more efficient enzymatic attack (Zhu and Pan, 2010). Pretreatment processes reported for wood biomass are dilute acid, sulphite treatment claiming to reduce the recalcitrance of the wood, steam explosion and organosolv (Zhu *et al*., 2009a).

## Hydrolytic enzymes for wood

Filamentous fungi are one of the main players in the lignocellulose degradation in natural environments being also the most important source of enzymes for commercial purposes. Fungi display a very efficient lignocellulose degradation machinery and the comprehensive analysis of this enzymatic system may facilitate the directed improvement of enzymatic cocktails suitable for the production of biofuels. Wood decay fungi and biofuel production share the same initial goal: to break down lignocellulose into its free monomers (primarily glucose, and also xylose, mannose, galactose, rhamnose and arabinose). Towards this end, wood decaying fungi have evolved a broad array of hydrolytic enzymes including cellulases, hemicellulases and pectinases, as well as oxidative enzymes serving diverse functions including depolymerization of lignin. These enzymes are known as carbohydrate‐active enzymes (CAZymes), and are classified into numerous families of glycoside hydrolases (GHs), glycosyl transferases (GTs), polysaccharide lyases (PLs), carbohydrate esterases (CEs) and auxiliary activities (AAs), the latter of which include secreted oxidative enzymes, such as cellobiose dehydrogenase (CDH) and lytic polysaccharide monooxygenase (LPMO, formerly GH61), implicated in polysaccharide degradation (Levasseur *et al*., 2013; Lombard *et al*., 2014). Besides this, there are others non‐hydrolytic cellulose active proteins (NHCAPs) that have been shown to enhance significantly cellulase‐driven plant cell wall hydrolysis at reduced protein loadings (Ekwe *et al*., 2013).

The ability of white‐rot fungi to degrade all polymeric components of plant cell walls, including lignin, can be explained by the presence in addition of haeme peroxidases with proposed roles in oxidative ligninolysis (PODs). Those enzymes include manganese peroxidases (MnP), lignin peroxidases (LiPs) and hybrid enzymes known as versatile peroxidases (VPs) (Ohm *et al*., 2014). Most of them also secrete laccases, whose genes were found to be upregulated in wood based media infected with white‐rot fungus. LiPs and VPs are able to oxidize and cleave the recalcitrant non‐phenolic structures that compose the bulk of lignin (Tien and Kirk, 1983; Hammel *et al*., 1993; Caramelo *et al*., 1999). MnPs and VPs oxidize Mn^2+^ to Mn^3+^, which can oxidize only the minor phenolic units in lignin (Gold *et al*., 2000). On the other hand, laccases cleave linkages in polymeric lignin and are capable of disrupting beta‐aryl ether bonds that represent the most dominant linkage in hardwood lignin and a critical step in lignin degradation (Camarero *et al*., 1994; Eggert *et al*., 1996).

The hemicellulose both in terms of monomers and their linkage composition is considerably more heterogeneous in comparison to cellulose. The precise composition of hemicellulose is strongly dependent on the plant species (Table 3). Based on the complexity of the hemicellulose oligosaccharides, i.e. the main monomers in branches, and the linkage composition and substitutions, the degradation of this prominent group of cell wall polysaccharides requires a greater diversity of enzymes. For example in hardwood degradation, xylanases, xyloglucanases and beta‐xylosidases are involved in degradation of the main backbone. However, accessory enzymes such as 4‐O‐glucuronoyl methylesterases, arabinofuranosidases, alpha‐galactosidases and acetylxylan esterases could be key enzymes removing hemicellulosic fraction and increasing accessibility of cellulases to the cellulose fibres and improving the enzyme hydrolysis process. Furthermore, for softwood the main hemicellulases are mannanases and beta‐mannosidases, while the auxiliary enzymes are likely alpha‐galactosidases and acetylxylan esterases; these compositional differences condition the enzyme cocktail formulation for the degradation of different biomass stocks (Kolbusz *et al*., 2014). Acetylxylan esterases remove the O‐acetyl groups from position 2 and/or 3 on the beta‐D‐xylopyranosyl or mannopyranosyl residues of acetyl glucuronoxylan and glucomannan, the main hemicelluloses in secondary cell walls of hardwoods and softwoods respectively. However, the acetyl content is much lower in softwoods than in hardwoods (Teleman *et al*., 2000, 2003; Pawar *et al*., 2013). During saccharification, acetyl groups in xylans and mannans create steric hindrance which limits the extent of hydrolysis by reducing the binding of many hydrolytic enzymes. For example, the action of endoxylanases is partially or completely hindered by acetyl groups. Sugar yields of beta‐xylosidases, beta‐mannosidases and beta‐glucosidases are increased by the addition of suitable esterases, indicating that these hydrolytic enzymes cannot release acetylated terminal residues from hemicellulosic oligosaccharides. De‐acetylation of hemicelluloses is therefore a prerequisite for their saccharification, which in turn is important for opening the cellulose surface to cellulolytic enzymes (Vazquez *et al*., 2001; Selig *et al*., 2009; Zhang *et al*., 2011).

**Table 3:** Main hemicellulases necessary depending on the type of hemicellulose oligosaccharides

Hemicellulases	Linkage	Corn stover	Hardwood	Softwood
Xylanase	Endo‐β‐(1,4)‐Xyl	Yes	Yes	No
β‐xylosidase	β‐(1,4)‐Xyl	Yes	Yes	No
Xyloglucanase	Endo‐β‐(1,4)‐Glc	No	Yes	No
Arabinofuranosidase	ɑ‐(1,2)‐Ara, ɑ‐(1,3)Ara	Yes	Yes	No
Acetylxylan esterases	ɑ‐(1,2)‐Acetyl, ɑ‐(1,3)‐Acetyl	No	No	Yes
4‐O‐glucuronoyl methylesterases	ɑ‐(1,2)‐4‐O‐metil‐ɑ‐glucuronic	Yes	Yes	No
Fucosidase	ɑ‐(1,2)‐Fuc	No	Yes	No
ɑ‐galactosidase	ɑ‐(1,3)‐Gal, ɑ‐(1,6)‐Gal	No	Yes	Yes
ɑ‐xylosidase	ɑ‐(1,6)‐Xyl	No	Yes	No
Mannanase	Endo‐β‐(1,4)‐Glc‐Man	No	No	Yes
β‐mannosidase	β‐(1,4)‐Glc‐Man	No	No	Yes

The enzymatic machinery for hemicellulose degradation also appears to be different between different wood decay fungi. Genes for acetylxylan esterases (CE1), endo‐xylanases (GH10) and beta‐glucuronidases (GH79) involved in processing glucuronoxylans are more represented in white‐rot fungi relative to brown‐rot genomes (Ohm *et al*., 2014). Brown‐rot genome polypores lack of genes that encode xyloglucanases (GH74), while genes encoding 4‐O‐glucuronoyl methylesterases (CE15) were more abundant in white‐rot fungi. This enzyme likely modifies glucuronoyl acid residues in xylan and has been proposed to hydrolyse ester linkages between xylan glucuronic acid residues and phenyl propane residues in lignin (Spanikova and Biely, 2006). The CE15 enzymes could possibly enhance the substrate accessibility. Beta‐xylosidases were equally prevalent in white‐ and brown‐rot fungi.

Those differences could be explained in part by their substrate preferences, especially the prevalence of glucomannans in softwood versus glucuronoxylans in hardwood (Hori *et al*., 2013). In order to utilize complex substrates as nutrients, fungi upregulate genes encoding specific enzymes for the targeted deconstruction of the available substrates (Macdonald and Master, 2012; Glass *et al*., 2013). Recent insights of that genetic flexibility have been obtained working with the thermophilic fungus *Myceliophthora thermophila* as a model organism. This organism synthesizes a complete set of enzymes necessary for the breakdown of cellulose of different kinds of lignocellulosic feedstock into sugar precursors for biofuels and chemicals. Using the genome sequence as a reference, the exoproteome has been used to examine the lignocellulolytic enzymes produced by fungi when cultured in woody biomass [softwood Kraft (SWKP) and mechanical (SWMP) pulps, and hardwood Kraft and mechanical (HWMP) pulps] (Kolbusz *et al*., 2014). In the exoproteome of *M. thermophila* grown on pulp, the relative level of cellulose‐degrading enzymes was similar in softwood and hardwood. Softwood pulps, both mechanical and Kraft, elicited the highest secretion of mannanases involved in degradation of the most abundant polymers in softwood (galactoglucomannans and glucomannans).

Last but not at least, the heterogeneous composition, in terms of monomers and their linkages, between different woody biomass is also altered by the selected pretreatment therefore affecting to the enzyme formulation that maximizes yields. Considering one of the most accepted pretreatment processes, high temperature steam explosion with dilute acid, the hemicellulose is hydrolysed to a high extent, releasing xylose oligomers and other soluble sugars and acetyl groups (Jørgensen *et al*., 2007). This means that the cocktails need to be formulated according to the composition of the material resulting from the pretreatment process selected.

## Enzymatic hydrolysis performance of woody biomass at high substrate loadings

The lignocellulose‐to‐sugars process using cellulollytic enzymes consists in a pretreatment for removal lignin and hemicellulose, enzymatic hydrolysis of polysaccharides to sugar monomers, fermentation of sugars to ethanol and recovery of ethanol from the process stream. Despite significant advances towards the commercialization of the so‐called second‐generation bioethanol or other bio‐based products from agricultural wastes like corn stover, with several industrial plants started in 2014 (Peplow, 2014), an economically feasible enzymatic wood‐to‐biofuel process is yet lagging behind (Van Wyk, 2001; Sun and Cheng, 2002). The enzyme hydrolysis step has been identified as a major techno‐economical bottleneck in the entire wood‐to‐ethanol bioconversion process. Industrial enzymatic hydrolysis of lignocellulose has to operate at high solid loadings increasing sugar concentration and final ethanol content after fermentation to improve distillation yields, resulting a more economically feasible process.

Hydrolysis conducted at high‐solids loadings has several obstacles, e.g. high concentration of fibrous materials reduce the mass transfer rate; furthermore, substrate consistency leads to a high concentration of inhibitory substances causing severe end‐product inhibition effects.

The inhibitory effect of high consistency hydrolysis has been reported repeatedly and can be illustrated with the work by Xiao *et al*. (2009). Two substrates were used, unbleached hardwood pulp (UBHW) and organosolv pretreated poplar (OPP). UBHW has a cellulose content of approximately 80% with 19.6% xylan. The UBHW represents an ‘ideal’ pretreated wood substrate as it contains a minimal amount of lignin and other contaminants. OPP contains 87% cellulose with little xylan. The lignin content of OPP is slightly higher than that of UBHW. A mixture of cellulases (Celluclast 1.5L) and beta‐glucosidase (Novozym 188) at an enzyme loading of approximately 35 mg g^−1^ glucan of Celluclast and 20 mg g^−1^ glucan of Novozym 188 was used. At 20% substrate consistency, UBHW reached a cellulose‐to‐glucose conversion rate of ~84% after 96 h of incubation. While at 2% consistency it attained 100% cellulose‐to‐glucose conversion after 24 h of incubation. The lower conversion rate at 20% consistency compared to 2% is mainly due to the inhibition effects from the high glucose content in the hydrolyzate (Xiao *et al*., 2009).

The OPP was hydrolysed at both 2% and 20% substrate consistencies. After 60 h of enzymatic hydrolysis at 2%, a 100% conversion of cellulose‐to‐glucose was obtained. Hydrolysis of OPP at 20% substrate consistency yielded about 85% after 48 h. In direct comparison UBHW and OPP treatments reached a similar cellulose‐to‐glucose yield (84% versus 85%) after hydrolysing at high consistency for 96 h.

Fujii *et al*. (2009), compared the commercial cellulolytic cocktails of Hyper‐producing strains of *Acremonium cellulolyticus* (Acremonium cellulase, AC; Meiji Seika) and *Trichoderma reesei* (Accellerase 1000; Genencor, Rochester, NY, USA) against milled lignocellulosic materials: eucalyptus, Douglas fir and rice straw. The composition for eucalyptus was 40.0% glucan and 10.4% xylan; Douglas fir was 51.9% glucan and 13.2% mannan; and rice straw was 37.0% glucan and 13.7% xylan. Enzymatic hydrolysis was performed using an enzyme constituting 22.5 or 90.0 mg protein per gram of dry substrate. The reaction mixture was then incubated at 50°C for 72 h at 20% total solids. Regarding the characterization of the different cocktails used, the AC mixture showed twofold and 16‐fold increases in Filter Paper and beta‐glucosidase specific activities, respectively, compared with Accellerase 1000. However, xylanase, beta‐xylosidase and beta‐mannosidase specific activities for Acellerase 1000 were higher than AC. The commercial AC enzyme demonstrated a lower cellulase specific activity than Accellerase 1000. The mannan‐hydrolysing activity of AC was also 16‐fold higher than that of Accellerase 1000.

The saccharification ability of these enzymes was evaluated during time‐course hydrolysis of the three ball‐milled lignocellulosic materials. They found that Accellerase 1000 and AC produced the same amount of glucose from each material at 72 h. However, the AC (90 mg protein g^−1^ substrate or 22.5 mg g^−1^ substrate) glucose yield at 3 h for the three lignocellulosic materials was higher than the Accellerase 1000 glucose yield. Therefore, the conversion of cellulose‐to‐glucose by AC was faster than by Accellerase 1000; this may be caused by a higher cellulase activity of AC. Meanwhile, culture supernatant derived from *T. reesei* was tested it exhibited a twofold higher xylan‐hydrolysing activity and produced more xylose from eucalyptus and rice straw.

During hydrolysis of three materials using commercial enzymes, AC produced more glucose than Accellerase 1000, even though it exhibited a lower cellulase specific activity. Similar results were also obtained during the analysis of xylose production. A possible explanation of this result may be the differences in substrate composition. The substrate used for the measurement of cellulase activity was highly purified. However, lignocellulosic materials contain additional elements including lignin, which is known to inhibit the cellulose hydrolysing reaction. Therefore, Accellerase 1000 may be more sensitive to cellulase inhibitors, such as lignin, than AC (Fujii *et al*., 2009).

Pretreatment explosion (WEx) was used to obtain high conversion and release of sugars from loblolly pine (Rana *et al*., 2012). The major barrier identified in biorefineries based on woody materials has been the high lignin content and the crystallinity of cellulose. WEx pretreatment attacks the lignin structure which has been found to allow for low enzyme usage; no chemical is added to run the process except water and oxygen/air and no need to recover and recycle the added chemicals after the pretreatment. WEx can also operate at higher dry matter concentration (Ahring and Munck, 2009).

Pretreatment explosion was conducted at 25% (w/w) solids in the presence of oxygen at 6.5–7.2 bar, temperatures of 170–175°C and residence time from 20 to 22.5 min.

Loblolly pine has a chemical composition of: glucan 35.97%, xylan 7.54%, galactan 2.47%, arabinan 1.57%, mannan 8.15% and lignin 30.65%. Loblolly pine samples were submitted to the three different pretreatments in which oxygen pressure, reaction time and reaction temperature were varied and were designated as LP1 (6.5 bar, 22.5 min, 175°C), LP2 (7.2 bar, 22 min, 170°) and LP3 (7.2 bar, 20 min, 170°C). Enzyme hydrolysis of the above pretreated samples was performed using Cellic^®^ Ctec2 (60 mg protein g^−1^ cellulose) and Cellic^®^ Htec2 enzymes (10% of Ctec2) at 50°C for 72 h. A mixture of two of these enzymes was used to determine the convertibility of cellulose and hemicellulose into monomers. The most enzymatic hydrolysis studies are conducted at 2–20% total solid to prevent enzyme inhibition. However, this enzymatic hydrolysis was performed at 25% total solid; the highest sugar yield was achieved from the pretreated LP2 sample with 96% for cellulose and about 100% for hemicellulose conversion. This is the highest sugar yield reported for loblolly pine (Rana *et al*., 2012).

## Conclusions

In this article, several aspects regarding enzymatic hydrolysis of woody biomass have been reviewed. It is clear that pretreatment conditions modify key chemical components and their bonds, i.e. they affect the hemicellulose composition and lignin structure; the pretreatment directly impacts the required enzyme dose. Lignin is the major barrier to enzymatic saccharification of wood cellulose due to the non‐productive adsorption of enzymes. In addition, different types of enzymes are needed based on the complexity of hemicellulose oligosaccharides, i.e. the major monomers in the branches; and the linkage composition and substitutions. This fact is directly correlated with selected wood biomass species. Hardwoods have higher glucuronoxylan and xyloglucan content, therefore xylanase, beta‐xylosidase and xyloglucanase are the main hemicellulases required for the degradation of the hemicellulose backbone. However, in softwood the most abundant polymers are galactoglucomannans and glucomannans, therefore the main hemicellulases needed are mannanases and beta‐mannosidases. Accessory enzymes such as 4‐O‐glucuronoyl methylesterases, arabinofuranosidases, alpha‐galactosidases and acetylxylan esterases are likely the key enzymes involved in removing the hemicellulosic fraction, and consequently increasing the accessibility of cellulases to the cellulose fibres, improving the hydrolysis process.

The enzyme hydrolysis step has been identified as a major techno‐economical bottleneck in the entire wood‐to‐ethanol bioconversion process. A diverse array of enzymatic cocktails has been tested, using high enzyme loading at 20–25% total solid and leading to a broad range of results. Overall, glucose yields between 60% and 95% are achieved with different pretreatments and diverse wood biomass species. In recent years a significant improvement in the commercially available cellulolytic cocktails has been achieved, however, the performance of these optimized cocktails on wood has not yet been reported in the literature.

## Funding information

No funding information provided.

## Conflict of interest

The authors declare that they do not have any conflict of interest for the publication of this article.
